# Micronutrients, *N*-Acetyl Cysteine, Probiotics and Prebiotics, a Review of Effectiveness in Reducing HIV Progression

**DOI:** 10.3390/nu2060626

**Published:** 2010-06-02

**Authors:** Ruben Hummelen, Jaimie Hemsworth, Gregor Reid

**Affiliations:** 1 Department of Public Health, Erasmus MC University Medical Center Rotterdam P.O. Box 2040, 3000 CA Rotterdam, The Netherlands; Email: r.hummelen@erasmusmc.nl; 2 Canadian Research & Development Centre for Probiotics, Lawson Health Research Institute, 268 Grosvenor Street, N6A 4V2, London, Ontario, Canada; 3 Division of Food and Nutritional Sciences at Brescia University College, The University of Western Ontario, 1285 Western Road, N6G 1H2, London, Ontario, Canada; Email: jaimie.hemsworth@lshtm.ac.uk; 4 Departments of Microbiology & Immunology and Surgery, The University of Western Ontario, 1151 Richmond Street, N6A 3K7, London, Ontario, Canada

**Keywords:** HIV, AIDS, micronutrients, zinc, selenium, N-acetyl cysteine, probiotics, prebiotics

## Abstract

Low serum concentrations of micronutrients, intestinal abnormalities, and an inflammatory state have been associated with HIV progression. These may be ameliorated by micronutrients, *N*-acetyl cysteine, probiotics, and prebiotics. This review aims to integrate the evidence from clinical trials of these interventions on the progression of HIV. Vitamin B, C, E, and folic acid have been shown to delay the progression of HIV. Supplementation with selenium, *N*-acetyl cysteine, probiotics, and prebiotics has considerable potential, but the evidence needs to be further substantiated. Vitamin A, iron, and zinc have been associated with adverse effects and caution is warranted for their use.

## 1. Introduction

Infections caused by the Human Immunodeficiency Virus (HIV) are one of the leading public health concerns around the world. Over 33 million people are living with HIV and nearly three million became infected during 2008 [1]. While antiretroviral therapies (ART) are almost universally available in developed countries, only since the G8 summit in 2005 have they been made more readily accessible to patients in developing countries [2]. As there is considerable risk for developing ART-induced toxic effects and metabolic dysfunction, the therapy is initiated only when the immune function is compromised (<350 CD4 cells/μL). For patients who have not developed severe immune deficiency, there is a significant void in methods to provide improved health. HIV causes certain dietary complications, including increased resting energy expenditure [3,4], enhanced oxidative stress [5], and a deleterious impact on the gastrointestinal system [6] that may require interventions specifically targeted to HIV. Therefore, the provision of a balanced and adequate diet is of prime importance for people living with HIV [7]. 

Vitamins and minerals (referred to as micronutrients) have received widespread and recent attention as potential interventions to delay HIV progression [8]. Although micronutrient interventions for people living with HIV have been thoroughly reviewed [9], to our knowledge, no review articles have integrated the evidence of the potential of various bioactive components, such as *N*-acetyl cysteine, probiotics, and prebiotics with micronutrients for this population. These interventions could potentially delay the progression of HIV and therefore postpone the moment that a patient becomes immune-compromised, and thus eligible for ART. Moreover, they could act in a complementary fashion with the ART, once the latter is initiated. Therefore, we present an assessment of randomized controlled trials (RCT) that evaluates the impact of micronutrients, *N*-acetyl cysteine, probiotics, and prebiotics on mortality, CD4 count, or HIV viral load among people living with HIV. Although this review emphasizes results from trials, it also briefly explores results from observational studies. 

## 2. Micronutrients

The use of micronutrients among people living with HIV is widespread and comes from the concept that the virus causes a diverse range of nutrient abnormalities (reviewed in [10]). The depletion of micronutrients may occur through increased metabolic requirements, enhanced excretion, and intestinal mal-absorption (reviewed in [11]). 

Early studies of micronutrients tested the effect of single vitamins or minerals on delaying the progression of HIV. While some successes were reported, the focus has now turned to multivitamin/mineral supplementation as a more comprehensive approach to delaying HIV progression. The levels of micronutrients consumed by people living with HIV are often much higher than recommended by the Dietary Reference Intakes, potentially leading to adverse events [12]. Therefore, health care professionals should be aware of the beneficial and potential adverse effects of micronutrients in order to make informed, safe, and practical decisions for people living with HIV. 

## 3. Vitamin A

Vitamin A is essential for normal immune function, the maintenance of mucosal surfaces, and haematopoiesis [13]. Also, vitamin A supplementation is recognized for its ability to decrease morbidity and mortality rates for some infectious diseases [14]. Vitamin A deficiency has consistently been associated with an increase in mother-to-child transmission of HIV (MTCT) [15]. However, there is conflicting evidence coming from observational studies on HIV progression and vitamin A where in one study, a cohort (n = 179) of HIV infected drug users, reported increases in mortality and lower CD4 counts associated with lower serum vitamin A levels [16]. In contrast, another observational study of HIV-infected men (n = 311) did not find a relationship between lower serum retinol (vitamin A) status and HIV disease progression or CD4 count [17]. 

There seems to be a potential role for vitamin A in children living with HIV. During an RCT in Tanzania, 687 children without signs of vitamin A deficiency who were admitted to the hospital with pneumonia were randomized. Some were given a large dose of vitamin A (400,000 IU) at baseline and again at four and eight months post-discharge. Among those, 58 were diagnosed with HIV and naïve to ART. Overall, a 49% reduction in mortality (p = 0.05) and a 92% reduction in diarrhea-related death (p = 0.01) was observed. These effects were more profound among the sub-group of children with HIV with a reduction in mortality of 53% (p = 0.04) and a reduction of 68% in AIDS related deaths (p = 0.05) [18]. Another RCT among 181 ART naïve HIV-infected children (15–36 months) in Uganda found similar results. Vitamin A supplementation (60 mg retinol) at three month intervals for a median of 17.8 months reduced mortality rates by 46% (p = 0.04), as well as persistent cough and chronic diarrhea. No effects were noted on CD4 count or HIV viral load [19]. Thus, there are potential benefits conferred by vitamin A supplementation for reducing the morbidity and mortality of children living with HIV in developing countries. 

Women living with HIV are at increased risk for experiencing adverse pregnancy outcomes, such as preterm birth and the delivery of low-birth-weight infants. Moreover, women can transmit the virus to their newborns through intrauterine, delivery, or breastfeeding routes. Research on the role of vitamin A in birth outcomes is critical as low levels of the vitamin have been associated with adverse pregnancy outcomes and vertical transmission of HIV [15]. To evaluate the effect of vitamin A supplementation on MTCT, RCTs were conducted among ART naïve pregnant women in South Africa, Malawi, and Tanzania. In South Africa, an RCT among 728 pregnant women showed that daily Vitamin A supplementation (5,000 IU and 30 mg β-carotene) and a large dose at delivery (200,000 IU) did not reduce MTCT or mortality among women and infants, although among those infants born pre-term, vitamin A was associated with a reduction in MTCT [20]. In the Malawian RCT, pregnant women with HIV (n = 697) were randomized to receive daily vitamin A (3 mg retinol) or placebo. Infants whose mothers were in the vitamin A group had a higher birth weight (p = 0.05) and fewer incidences of anemia at six weeks, from 41% to 23% (p = < 0.001). This trial, however, did not show vitamin A supplementation to have an effect on MTCT [21].

Another trial among HIV-infected pregnant women in Tanzania (n = 1,078) did not show any effects of vitamin A supplementation (30 mg β-carotene plus 5000 IU vitamin A) on improved pregnancy outcomes. In contrast, vitamin A supplementation appeared to increase the MTCT of HIV by an additional 38% compared to the placebo (p = 0.009) [22]. Furthermore, vitamin A did not have any effect on HIV-associated mortality or CD4 count during a median 71 months of follow-up among the women [23,24]. 

In conclusion, two studies have indicated a potential benefit of vitamin A supplementation for children with HIV in developing countries who are naïve to ART. However, these benefits need to be confirmed in larger RCTs with HIV progression, mortality, and morbidity as primary outcomes. With respect to maternal supplementation, little evidence exists to support the use of vitamin A supplements in HIV-positive pregnant women. Rather, the possibility that maternal vitamin A supplementation could increase the risk of vertical transmission warrants caution for its use among this population. The notion that vitamin A supplementation reduced the benefits of multivitamin supplementation indicates that no profound benefits may exist for supplementing vitamin A among adults.

**Table 1:** Randomized controlled trials on vitamin A and HIV progression.

Reference	Population, in- and exclusion criteria.	Intervention and follow-up	Primary outcomes	Major findings	Conclusions
Fawzi 1998 [22,23,24]	Tanzania, 1078 ART naïve pregnant women.	Multifactorial design with Vit. A (5000 IU) and β-carotene (30 mg) daily during pregnancy and lactation.	Mortality, CD4 count and viral load.	No differences in mortality, CD4 count or viral load among women or children. Vit. A increased MCTC.	Vit. A does not reduce mortality among women but increased MTCT.
Fawzi 1999 [18]	Tanzania, 58 ART naïve children admitted with pneumonia.	Dose vit. A (400,000 IU) at baseline, 4 and 8 months.	Mortality	Reduced overall and AIDS related mortality. Reduction on diarrhea related death.	Vit. A reduces mortality among children admitted with acute infections.
Coutsoudis 1999 [20]	South-Africa, 728 ART naïve pregnant women.	Vit. A (5000 IU), β-carotene (30 mg) third trimester and vit. A (200,000 IU) at delivery.	MCTC, fetal and infant mortality.	No reduction in MCTC, fetal or infant mortality. Reduction in preterm delivery.	Vit. A administered to the mother does not affect fetal or infant mortality or MCTC.
Kumwenda 2002 [21]	Malawi, 697 ART naïve pregnant women.	Vit. A (3 mg) daily from 18-28 weeks of gestation until delivery.	MTCT, birth weight.	Increase in birth weight and a reduction of the number of anemic children. No effect on MTCT.	Vit. An administered to the mother increases birth weight and prevents anemia. No effect on MTCT
Semba 2005 [19]	Uganda, 181 ART naïve children.	Vit. A (60 mg) every three months for 18 months.	Mortality, CD4 count, HIV viral load.	Reduction in mortality. No effect on CD4 count or viral load.	Vit. A reduces mortality among children.

MTCT = mother to child transmission

## 4. Vitamin B, C, E and Folic Acid

B-vitamins are essential components of our immune system. Riboflavin deficiency hampers the generation of a humoral antibody response, vitamin B-6 deficiency reduces the maturation of lymphocytes, and vitamin B-12 deficiency impairs the function of neutrophils [25]. It is therefore not surprising that among people living with HIV, higher serum levels and intake of B vitamins have been associated with improved survival. In a large cohort study of people living with HIV in the US (n = 241), the highest quartile of intake for each B-group vitamin was independently associated with improved survival over the course of an eight-year follow-up: B1, (relative hazard (RH) = 0.60), B2 (RH = 0.59), B6, (RH = 0.45), and niacin (RH = 0.57) [26]. Higher serum levels of vitamin B12 were also associated with delayed HIV progression [27]. These results were confirmed in a matched case-control study within a cohort of South African HIV patients. The median survival time of patients using vitamin B supplements (264 weeks) was significantly longer than of those not taking B vitamins (144 weeks) (p = 0.0014) [28]. 

In several observational studies, vitamins C and E were found to be lower among people living with HIV [5,10]. These findings were confirmed by the group of Baum *et al*., who compared the nutrient levels of 108 HIV-infected gay men, almost all with high CD4 counts, to a similar group of HIV-negative controls. In this seminal study, they found that deficiencies of vitamins B6, B12, A, E, and zinc were highly prevalent in the HIV-infected cohort. Thus, the conclusion was that HIV-infected individuals most likely require micronutrient intake in multiples of the DRI in order to maintain normal serum levels [29]. Lower levels of vitamin C and E were also related to significantly higher levels of oxidative stress [5], which presumably leads to increased viral replication [30]. In longitudinal studies, the dietary intake of vitamin E [31] and higher vitamin E serum levels [17] were associated with higher CD4 counts and a reduced risk of HIV progression [32]. 

These findings were the impetus for studies assessing whether exogenous supplementation of vitamins could reduce oxidative stress and therefore potentially reduce HIV viral load. A pilot RCT (n = 49) among HIV patients receiving ART showed that the group receiving vitamin E (800 IU) and C (1,000 mg) for three months had significantly less oxidative stress (reduction of plasma peroxides and malondialdehyde) and a trend was seen (p = 0.1) towards a reduction of the viral load (−0.45 log^10^ copies/ml) compared to the placebo group (*+*0.50 log ^10^ copies/mL) [33]. A study in Uganda with a short follow-up period of six weeks did not find a benefit of supplementation. The RCT (n = 141) among ART naïve HIV patients admitted with diarrhea showed that supplementation with a combination of vitamin A (10,500 IU), E (300 mg), Selenium (150 μg), and Zinc (200 mg) for two weeks did not reduce the length of time with diarrhea. Moreover, no effects were seen on mortality or CD4 count after the six weeks of follow-up. Low serum levels of vitamin A and E were confirmed to be predictors of mortality in this study, but exogenous supplementation did not increase these levels [32]. The expectation to see an effect of supplementation within such a short time-frame might have been unrealistic, as an impact on HIV progression is typically assessed over longer periods of time.

The first long term study with HIV progression and mortality as primary outcome was conducted in Tanzania. A specific multivitamin combination of vitamins B, C, E, and folic acid (20 mg B_1_, 20 mg B_2_, 25 mg B_6_, 100 mg B3, 50 μg B12, 500mg C, 0.8mg folic acid) was repeatedly shown to increase CD4 and CD8 counts, lower viral loads, and reduce mortality among ART naïve HIV infected women [34]. The large RCT (n = 1075) among pregnant women showed an increase in CD4 count from baseline (between 12 and 27 weeks gestation) to six weeks postpartum of 176 cells/μL *vs.* 112 cells/μL (p = < 0.001) in the placebo group. At 30 weeks postpartum, there was still a significant difference with an increase of 99 CD4 cells/μL in the multivitamin group *vs.* an increase of 59 CD4 cells/μL in the placebo group (p = 0.05), in addition to an increase in CD8 and CD3 cells. Furthermore, there were significant reductions in fetal death (Relative Risk (RR) = 0.61), low birth weight (RR = 0.56), and severe preterm birth (RR = 0.61) with multivitamin supplementation [34]. Long term (71 months) follow-up of this cohort showed that only 67 of 271 (25%) women receiving multivitamins died or developed stage 4 disease compared to 83 of 276 (30%) receiving placebo (RR = 0.71, P = 0.04). Moreover, women receiving multivitamin had on average a CD4 count of 48 cells/μL higher than the placebo group (p = 0.01), and a significantly lower viral load (p = 0.02) [24]. Multivitamins also reduced the risk of wasting (RR = 0.66, p = 0.02) and the incidence of weight loss episodes amongst HIV infected women [35]. Interestingly, women with impaired immunological (low lymphocyte count) and nutritional status (low haemoglobin levels) at baseline had reduced transmission of the virus from mother to child (RR = 0.37, p = 0.02) and decreased child mortality after 24 months (RR = 0.82, p = 0.08) when given multivitamins [22]. These results suggest that this particular multivitamin supplement (20 mg B_1_, 20 mg B_2_, 25 mg B_6_, 100 mg B3, 50 μg B12, 500mg C, 0.8 mg folic acid) can be safely administered to pregnant women and has considerable immunological benefits. 

To assess the impact of the same multivitamin formula and selenium (200 μg daily for six weeks) on the infectivity (vaginal HIV shedding) of ART naïve women with HIV, an RCT (n = 400) was launched in Kenya. Although a relatively short intervention period of six weeks was used and the primary outcome was infectivity, the study confirmed the beneficial effects of the multivitamin supplement on the CD4 count (a mean of 23 cells/μL higher than placebo) and CD8 count (a mean of 74 cells/μL higher than placebo). However, the trial showed a potential increase of HIV infectivity amongst women receiving supplements as the odds of detecting HIV infected vaginal epithelial cells increased 2.5 fold (p = 0.001), as well as the quantity of vaginal HIV RNA (p = 0.004) [36]. 

In Bangkok, an RCT (n = 481) was conducted with an intervention using similar concentrations of vitamin B, C, E, and folic acid (24 mg B_1_, 15 mg B_2_, 40 mg B_6_, 30 μg B12, 400mg C, 3 mg A, 0.1 mg folic acid), but with the addition of vitamin A, zinc, and various other micronutrients (table 2.). Overall, a trend was seen towards a reduced mortality in the multivitamin group (RR = 0.53, p = 0.1) and a significant reduction among subgroups of those with a CD4 count <200 (RR = 0.37, p = 0.05) and <100 (RR = 0.26, p = 0.03). However, no effect was seen on viral load and CD4 count [37]. 

A multivitamin combining vitamins A, B, C, D, E, zinc, copper, selenium, iodine, and folic acid with concentrations approximately 10 times lower than the Tanzanian combination was tested within a cluster randomized trial in Zambia. Households (n = 500 individuals) were randomized to receive the multivitamin combination for an average of 3.3 years [45]. No differences were seen in terms of CD4 count or diarrhea incidence amongst those living with HIV who are ART naïve (n = 135). However, a significant difference in mortality was reported, as 20% (12/61) died during follow-up with placebo as compared to 5% (4/74) amongst the multivitamin supplemented group (p = 0.03 by log-rank test).

In the United States, an RCT (n = 40) among HIV patients treated with ART who displayed related neurotoxicity, tested the impact of an intervention combining vitamins B, C, E, and folic acid in a daily dose approximately 6 times higher than the Tanzanian multivitamin combination and with the addition of vitamins A, D, zinc, *N*-acetyl cysteine (1.2 g), and various other minerals (Table 2). The intervention group experienced an increase in CD4 count from a baseline of 65 cells/μL after 12 weeks of supplementation, as compared to −6 cells/μL in the placebo group. However, no differences were seen in viral load or neuropathy scores [38].

In summary, several well designed RCTs have shown a considerable benefit of supplementation of vitamins B, C, E, and folic acid (20 mg B_1_, 20 mg B_2_, 25 mg B_6_, 100 mg B3, 50 μg B12, 500 mg C, 0.8 mg folic acid) to ART naïve HIV patients. Although in subpopulations a decrease in MTCT has been reported, further studies are needed to assess if the potential increase in infectivity is present and may enhance HIV transmission. Furthermore, as one trial has shown potential for this approach among an ART-treated population, these results need to be confirmed by larger RCTs with long term follow-up. 

## 5. Vitamin D

The interest in vitamin D and the immune system has been catalyzed by the discovery of vitamin D receptors in peripheral blood mononuclear cells involved in immune system regulation [39]. Vitamin D is involved in immune-related diseases, such as Ulcerative Colitis and Crohn’s Disease, and has recently gained attention for its mediating role in innate immunity and the body’s response to intracellular micro-organisms, such as *Mycobacterium tuberculosis* [40]. The role of vitamin D in HIV progression is less well understood. In Tanzania, an observational study (n = 1,078) was conducted within a trial to assess the effects of the vitamin D status of pregnant women on adverse pregnancy outcomes, MTCT, and child mortality [41]. Children born to mothers with low vitamin D levels had a 64% greater risk of dying during follow-up and an overall 46% higher risk of contracting HIV. In this observational study of vitamin D status’ effects on child morbidity and mortality, a non-linear relationship was found between vitamin D levels and MTCT (p = 0.01), where the risk of MTCT decreased as the levels of vitamin D increased [41]. The only RCT with findings on vitamin D and HIV progression to date was conducted in Guinea-Bissau. The trial (n = 367) among Tuberculosis (TB) patients assessed the impact of supplements of vitamin D every four months (100,000 IU of oral cholecalciferol) over a 12-month period [42] on mortality and the clinical severity of TB (Table 2). Vitamin D did not appear to improve the severity of tuberculosis, nor was there an impact on mortality in both the complete group and the sub-group infected with HIV (n = 135) [42]. Therefore, there is insufficient evidence to recommend vitamin D use among people living with HIV. Larger RCTs are warranted, as there might be considerable potential.

**Table 2:** Randomized controlled trials on vitamin B, C, D, E and folic acid, and HIV progression.

Reference	Population, in- and exclusion criteria.	Intervention and follow-up	Primary outcomes	Major findings	Conclusions
Allard 1998 [35]	Canada, 49 individuals receiving ART.	Daily vit. E (800 IU) and C (1000mg) for 3 months.	HIV viral load, oxidative stress	Trend towards reduction in viral load. Significant reduction of oxidative stress.	Vit. E and C may reduce viral load and reduces oxidative stress.
Kelly 1999 [34]	Zambia, 141 ART naïve patients admitted with diarrhea.	Daily vit. A (10.500 IU), C (300 mg), E (300 mg) Selenium (300 μg) and Zinc (200 mg) for two weeks.	Diarrhea, mortality, serum A and E, CD4 count.	No difference in length of diarrheal episodes, mortality or CD4 count after 6 weeks.	No additional value of multivitamin to treatment of diarrhea among HIV patients.
Fawzi 1998.[22,24,43]	Tanzania, 1078 ART naïve pregnant women.	Multifactorial design with Vit. B, C, E and folic acid^1^.	MTCT, Mortality, CD4 count and viral load.	No effect on MTCT, Reduced mortality, delayed HIV progression, increased CD4, CD8 count and decreased viral load for the multivitamin.	Multivitamin does not reduce MTCT but reduces HIV progression and mortality among children and pregnant women.
Jiamton 2003 [44]	Bangkok, 481 ART naïve individuals with CD4 count 50 - 500 cells/μL.	Daily vit. A, B, C, D, E, K, zinc, selenium and various minerals^2^ for 48 weeks.	Mortality, CD4 count	Reduction of mortality with no differences in viral load and CD4 count.	Micronutrients reduce mortality among people living with HIV.
McClelland 2004 [36]	Kenya, 400 ART naïve women.	Daily vit. B, C, E, folic acid^1^ and selenium (200 mg) for 6 weeks.	HIV infectivity	Increase in HIV infectivity. Increase in CD4 and CD8 count and no difference in viral load.	Multivitamin may increase HIV infectivity.
Kaiser 2006 [38]	United states, 40 individuals receiving ART and signs of ART related neuropathy.	Twice daily vit. A, B, C, D, E, zinc, selenium, NAC and various minerals^3^ for 12 weeks.	CD4 count, viral load, neuropathy, safety parameters.	Intervention increased CD4 count with no effect on viral load or neuropathy.	Micronutrients and NAC increases CD4 count and was found to be safe.
Kelly 2008 [45]	Uganda, cluster randomized trial among 500 individuals of which 135 HIV infected and ART naïve.	Daily vit.A, B, C, D, E, folic acid, selenium, iron, zinc, copper, iodine^4^.	Diarrhea incidence, CD4 count, mortality.	Intervention did not reduce the incidence of diarrhea. No effect on CD4 count but decrease in mortality.	Low dose multivitamin may reduce mortality.
Wejse 2009 [42]	Guineau-Bassau, West Africa, 367 TB-infected, 131 co-infected with HIV and ART naïve.	Vitamin D 100,000 IU every 3 months for 12 months	Clinical severity score of TB and overall mortality at 12 months	No difference in clinical severity or mortality between vitamin D and control group.	Vit. D not effective at improving clinical outcomes of TB.

MTCT = Mother to child transmission, TB = Tuberculosis

1. Micronutrient supplement included vitamin B1 20 mg , vitamin B2 20 mg, vitamin B6 25 mg, vitamin B3 100 mg, vitamin B12 50 μg, vitamin C 500 mg and folic acid 0.8 mg.

2. Micronutrient supplement included vitamin A 3,000 μg, beta-carotene 6 mg, vitamin D3 20 μ g, vitamin E 80 mg, vitamin K 180 μg, vitamin C 400 mg, vitamin B1 24 mg, vitamin B2 15 mg, vitamin B6 40 mg, vitamin B12 30 μg, folacin 100 μ g, panthothenic acid 40 mg, iron 10 mg, magnesium 200 mg, manganese 8 mg, zinc 30 mg, iodine 300 μg, copper 3 mg, selenium 400 μg, chromium 150 μg and cystine 66 mg.

3. Micronutrient supplement included *N*-acetyl cysteine 1,200 mg , acetyl L-carnitine 1,000 mg, α-lipoic acid 400 mg, β-carotene 20,000 IU, vitamin A 8,000 IU, vitamin C 1,800 mg, thiamine 60 mg , riboflavin 60 mg, pantothenic acid 60 mg, niacinamide 60 mg, inositol 60 mg, vitamin B6 260 mg, vitamin B12 2.5 mg, vitamin D 400 IU, vitamin E 800 IU, folic acid 800 μg, Ca 800 mg, Mg 400 mg, Se 200 μg, Iodine 150 μg, Zn 30 mg, Cu 2 mg, B 2 mg, K 99 mg, Fe 18 mg, Mn 10 mg, biotin 50 μg, Cr 100 μg, Mo 300 μg, choline 60 mg, bioflavonoid complex 300 mg, L-glutamine 100 mg, and betaine HCL 150 mg.

4. Micronutrient supplement included Vitamin A as β-carotene 4,8 mg, vitamin B1 1,4 mg , vitamin B2 1,4 mg, vitamin B6 1,6 mg, vitamin B12 2,6 μg, vitamin C 18 mg, vitamin D35 mg, vitamin E 10 mg, iron 30 mg, zinc 15 mg, copper 12 mg, selenium 65 μg iodine 150 μg and folic acid 400 μg.

## 6. Iron

Iron deficiency is the most common nutritional deficiency in the world, and is a condition mainly affecting women. Among people living with HIV, anemia (defined as low haemoglobin) is highly prevalent and is associated with increased mortality [46,47] and enhanced HIV progression [47]. Although anemia can generally be treated by iron supplementation, various clinical observations have raised the concern that iron supplementation may adversely affect HIV progression and increase mortality [48]. Firstly, in a retrospective study among thalassemia major patients (n = 64), the rate of progression of HIV was significantly faster amongst those receiving lower doses of the iron chelating agent, desferrioxamine, and who have higher serum ferritin concentrations [49,50]. Second, the concurrent administration of low doses of iron with dapsone for the prophylaxis of *Pneumocystis carinii* pneumonia in HIV-positive patients was associated with an increased risk of mortality in a randomized study among 196 HIV patients [51]. In contrast, the administration of Dapsone for the prophylaxis of *Pneumocystis carinii* without a low dose of iron did not lead to excess mortality in another trial [52]. Thirdly, a study on polymorphisms of haemoglobin-binding haptoglobin indicated that the haptoglobin 2-2 polymorphism is associated with higher iron stores and shortened survival among HIV patients [53]. Finally, a retrospective study of iron load in bone marrow macrophages among HIV patients suggested that high iron stores were associated with a shorter survival in HIV-positive patients [54]. As none of these observations could provide conclusive evidence for an adverse effect of iron on HIV progression, two prospective studies were conducted. Within a larger RCT of non-anemic participants (n = 181), a subpopulation of ART naïve HIV patients (n = 45) was studied to assess the effect of daily 60 mg iron supplementation (twice weekly for 4 months) on HIV viral load (Table 3). In this small study, no increase in HIV viral load could be detected [55]. In the United States, an RCT was conducted among female drug users infected with Hepatitis C Virus (n = 485) and among them, a subgroup co-infected with HIV (n = 138), of whom 50 were treated with ART. An assessment was made on whether iron supplementation (18 mg daily) would increase HIV viral load and reduce anemia over a 12-month period. At follow-ups, the proportion with anemia in the iron group was only 21% *vs.* 31% in the placebo group (P = 0.03) at 6 months and, respectively, 26% *versus* 30% (P = 0.5) at 12 months. Furthermore, no increase in HIV viral load could be detected [56]. This study could exclude a large increase in HIV viral load due to iron supplementation, but was not powered to detect small increases over a larger period of time. Furthermore, the conclusions from this study cannot readily be extrapolated to other HIV populations. It remains to be determined what the influence is of iron supplementation among patients who are receiving ART. Given the potential adverse role of iron in HIV progression, caution is warranted for iron supplementation among HIV infected populations. Routine supplementation without a specific indication is contra-indicated.

**Table 3:** Randomized controlled trials on iron supplementation and HIV progression.

Reference	Population, in- and exclusion criteria	Intervention and follow-up	Primary outcomes	Major findings	Conclusions
Olsen 2004 [55]	Kenya, 45 ART naïve HIV patients.	60 mg iron twice weekly for 4 months.	HIV viral load.	No increase in HIV viral load.	Low dose iron can be safely administered.
Semba 2007 [56]	United States, 485 HCV infected of which 138 co-infected with HIV of whom 50 receiving ART.	18 mg iron daily for 12 months.	HCV and HIV viral load, anemia.	No increase in HCV or HIV viral load. Reduced occurrence of anemia.	Iron is safe administered and is effective in treating anemia.

HCV = Hepatitis C Virus.

## 7. Zinc

Various studies have investigated the role of zinc in HIV disease progression. The immunologic consequences of zinc deficiency are manifold, given the involvement of the mineral in basic cellular processes, which are essential in immunological mechanisms [57]. Consequently, zinc deficiency has been associated with a higher susceptibility to infectious diseases, such as pneumonia, malaria, and diarrhea [58]. There is evidence that zinc deficiency amongst people living with HIV may account for an improper maturation of CD4 count cells mediated through low levels of the zinc-dependent hormone, thymuline. This may lead to a less effective immune response and a higher susceptibility to opportunistic infections [59]. However, evidence for the relationship between zinc status and HIV is inconclusive. In an observational study of asymptomatic HIV-infected men in the United States, high zinc intake has been shown to be significantly associated with faster HIV progression and an increased mortality amongst men [17,60]. In contrast, another observational US cohort study of HIV positive men suggested that higher serum zinc levels were inversely associated with mortality [61]. 

A first interventional study was conducted in Italy among 57 Zidovudine treated HIV patients that had developed or were at risk of developing AIDS (Table 4.). Those supplemented daily with zinc (45 mg) for 30 days experienced, at follow-up, a lower frequency of opportunistic infections after 24 months and an increase in CD4 count after four months (compared to the controls) [59]. To assess the safety of daily zinc supplementation (10 mg) to ART naïve children with HIV, an RCT (n = 96) was conducted in South Africa. This trial did not show any apparent benefit of zinc supplementation. No differences were found in CD4 count, viral load, or haemoglobin concentration, while supplementation was found to be safe [62]. This trial was followed by the largest study to date on zinc supplementation for people living with HIV and was aimed at assessing the potential benefits of zinc supplementation on pregnancy outcomes. The study population of the RCT (n = 400) consisted of pregnant ART naïve women with HIV in Tanzania [63]. All women received the multivitamin supplement (discussed in the section on vitamins B, C, E, and folic acid) and were randomized to receive, in addition, either a placebo or a 25 mg daily dosage of zinc between recruitment and six weeks after delivery. The results showed no differences in pregnancy outcomes, HIV transmission, CD4 or CD8 count, and viral load. However, zinc supplementation was inversely associated with haemoglobin levels, and was related to a threefold increase in the probability of wasting (RR = 2.7, p = 0.03) [64]. 

In conclusion, although an initial trial has shown potential benefits of zinc supplementation among ART treated patients, these results have not been replicated in a trial among ART naïve pregnant women. Further research is warranted to assess whether there is a potential role for zinc among HIV patients treated with ART. 

**Table 4:** Randomized controlled trials on zinc and HIV progression.

Reference	Population, in- and exclusion criteria	Intervention and follow-up	Primary outcomes	Major findings	Conclusions
Mocchegiani 1995 [59]	Italy, 57 AZT treated patients. At risk or developed AIDS.	Zinc (45 mg) for 30 days.	Incidence opportunistic infections, body weight.	Reduction opportunistic infections, stabilization or increase body weight, CD4 count, thymulin.	Zinc increases CD4 count.
Bobat 2005 [62]	South Africa, 96 ART naïve children.	Daily zinc (10 mg) for 6 months.	CD4 count, viral load and diarrhea incidence.	No difference in CD4 count or viral load but a reduction in watery diarrhea.	Zinc safe among children with HIV.
Fawzi 2005 [63,64]	Tanzania, 400 ART naïve pregnant women.	Daily zinc (25 mg) additional to multivitamin ^1 ^until 6 weeks after delivery.	CD4 count, viral load and MTCT.	No effects on CD4 count, viral load and MCTC but adverse effects on haemoglobin level and an increase risk of wasting.	Zinc has no impact on HIV progression and may cause adverse effects.

MTCT = Mother to child transmission; 1. Micronutrient supplement included vitamin B1 20 mg , vitamin B2 20 mg, vitamin B6 25 mg, vitamin B3 100 mg, vitamin B12 50 μg, vitamin C 500 mg and folic acid 0.8 mg.

## 8. Selenium

The trace element selenium has been suggested to be a key nutrient for people living with HIV. Lower serum concentrations of selenium in both adults and children infected with HIV have been linked to increased mortality [65] and an increased viral load [66,67,68]. The role of selenium in immunity and antioxidant defence may be the underlying mechanism of an enhanced HIV progression with lower levels of the micronutrient [69]. These observational findings instigated clinical trials to assess whether selenium supplementation could have an impact on HIV viral load, CD4 counts, or HIV progression (Table 5). An earlier pilot RCT among ART treated HIV patients (n = 52) showed that selenium supplementation (100 μg) could enhance the antioxidant status of HIV patients [70]. A larger RCT (n = 186, of which 124 were receiving ART) showed that daily supplementation with selenium (200 μg) did not affect CD4 count levels or viral load after two years of follow-up. However, a smaller proportion (25%) among the selenium group experienced a substantial decline in CD4 count (>50 cells/μL) when compared to the placebo group (46%, p = 0.01). In addition, the relative risk of being admitted at a hospital was 2.4-times lower among the selenium supplemented group in comparison to the placebo group (p = 0.008) [71]. In a more recent RCT among 262 HIV-infected patients (192 receiving ART), nine-month supplementation with selenium (200 μg daily) resulted in an increase in CD4 count (p = 0.04) and a decrease in viral load (p = 0.02) [72]. 

To assess the impact of selenium supplementation on mother and child mortality, a large RCT (n = 913) was conducted amongst pregnant women in Tanzania. Daily selenium (200 μg) supplementation until six months post partum did not affect CD4 count, viral load, or overall mother and child mortality. However, at six weeks after delivery, a reduction of 57% in child mortality was found (p = 0.05) amongst the selenium supplemented group [73]. The authors concluded that the lack of a beneficial effect of selenium supplementation on HIV progression may have been due to the low prevalence of ART use and selenium deficiency amongst their study population or because of a reduced effect of selenium amongst patients already supplemented with micronutrients [73]. 

**Table 5:** Randomized, placebo controlled trials on selenium and HIV progression.

Reference	Population, in- and exclusion criteria	Intervention and follow-up	Primary outcomes	Major findings	Conclusions
Constans 1996 [70]	France, 52 individuals receiving ART.	Selenium (100 μg) *vs.*β-carotene (30 mg) *vs.* placebo	CD4 count and serum anti-oxidant level.	Both selenium and β-carotene did not have an impact on the CD4 count but enhanced serum antioxidant levels.	Selenium and β-carotene improve anti-oxidant status.
Burbano 2002 [71]	United states, 186 injection drug users of whom 124 receiving ART.	Daily Selenium (200 μg) for 2 years.	CD4 count and hospital admissions	No differences in CD4 count and viral load. Reduction in number with drop in CD4 count of >50 cells/μL and risk of admission was lower.	Selenium may reduce decline in CD4 count and risk of hospital admission.
Hurwitz2007 [72]	United states, 262 individuals of whom 192 receiving ART	Daily selenium (200 μg) for 9 months.	CD4 count, viral load.	Increase in CD4 count, and decrease in viral load.	Selenium reduces HIV progression.
Kupka 2008 [73]	Tanzania, 913 ART naïve pregnant women.	Daily selenium (200 μg) until 6 months after delivery.	Mortality, CD4 count, viral load.	No differences in mortality, CD4 count or viral load.	Selenium does not affect HIV progression.

At present, there is no support for providing selenium supplements to HIV patients who are not selenium deficient and who are already receiving a high-dose multivitamin. Although two RCTs have shown that selenium supplementation can potentially reduce the viral load and enhance the immunological status of patients treated with ARTs, well designed trials with a long term follow-up and an appropriate sample size among similar populations are needed to substantiate the evidence for the use of selenium.

## 9. N-acetyl Cysteine

Glutathione (GSH) is a principal regulator of oxidative stress, which occurs in the intracellular space and on cellular membranes. HIV-infected people tend to have abnormal GSH levels in various components of the immune-system, including CD4 cells. *In vitro* studies have shown that lower intracellular GSH levels decrease cell survival and increase NF-ĸB activation and consequently, HIV replication. Observational studies have linked GSH deficiency directly to patient survival [74]. Various interventions have been tested to increase GSH levels to normal among HIV patients. N-acetyl cysteine (NAC), a pro-drug used to replenish GSH after paracetamol intoxication, was shown to restore normal GSH levels among HIV patients [74,75,76]. 

One of the first RCTs was conducted by Herzenberg *et al*., who randomized HIV patients (n = 246, mixed ART status) with low GSH levels to NAC supplementation (3.2–8 g daily) or to a placebo for eight weeks, followed by an open label treatment of two years (Table 6). The results suggested that those using NAC were more likely to survive during follow-up (RR = 1.8, p = 0.02), but no CD4 count or viral load data were collected [74]. A smaller RCT (n = 45) among ART naïve HIV patients taking NAC supplementation (400 mg) for four months showed significantly reduced TNF-α levels, but no effect upon CD4 count during the study. However, it was noted that the NAC supplemented group had a less profound decline in CD4 count when compared to the 36 months before randomization (p = <0.05) [75]. Another RCT (n = 29) assessing the effect of daily NAC supplementation (0.6–3.6 g) during 7 months did not show differences in CD4 and CD8 counts, although an increase in natural killer (NK) cell activity (p = <0.05) was noted [76]. A controlled cross-over study (n = 24) showed a trend towards an increased CD4 count and CD4/CD8 ratio during daily supplementation of NAC (600 mg) when combined with selenium (500 μg) [77]. Serum GSH concentrations were not affected by supplementation. 

Another approach to replenishing GSH levels is through the supplementation of whey protein, which is a source of the amino acid, cysteine. This intervention was shown to be effective in enhancing GSH levels in a small RCT amongst patients receiving ART (n = 30) [78]. The effectiveness of whey protein on CD4 count levels was assessed in a larger RCT, which was also amongst ART treated patients (n = 59). In this study, the whey supplemented group (40 g daily) had an increase of CD4 count of 31 cells/μL, compared to 15 cells/μL amongst the placebo group during the 12-week follow-up (p = 0.03) [79]. 

Replenishing GSH stores with either NAC or whey protein appears to have a beneficial impact upon the immune function of people living with HIV. These results are promising and warrant the conduct of a large RCT to assess either of the effects of these supplements upon the progression of HIV. 

**Table 6:** Randomized, placebo controlled trial on cysteine and HIV progression.

Reference	Population, in- and exclusion criteria	Intervention and follow-up	Primary outcomes	Major findings	Conclusions
Akerlund 1991 [75]	Sweden, 45 ART naïve individuals with CD4 > 200 cells/uL	Daily NAC (400 mg) for 4 months.	CD4 count and clinical signs and symptoms	No effect on CD4 count. But increased level of GSH, reduced TNFα levels and reduced decline of CD4 count than before baseline.	NAC may reduce decline in CD4 count and restores GSH levels.
Herzenberg1997 [74]	United states, 246 individuals with low glutathione levels (ART status unknown).	Daily NAC (3.2–8.0 g) for 8 weeks, followed by open label treatment for 2 years.	Mortality, GSH levels.	Supplementation restored GSH levels and reduced mortality.	NAC restores GSH levels and may reduce mortality.
Look 1998 [77]	Germany, Cross-over RCT, 24 ART naïve individuals.	Daily NAC (0.6 g) and selenium (500 μg) for 24 weeks.	CD4 count, GSH levels	Trend towards increase in CD4 count and CD4/CD8 ratio at particular time points, no increase in GSH level.	NAC and selenium may improve immune status.
Breitkreutz 2000 [76]	Germany, 29 ART naïve individuals.	Daily NAC (0.6–3.6 g) for 7 months.	CD4 count, GSH levels	Supplementation restored GSH levels, no difference in CD4 or CD8 cells but increase in NK cell activity.	NAC restores GSH levels and may enhance antiviral response.
Micke 2002 [78]	Germany, 30 individuals receiving ART.	Daily whey protein (45 g) for 6 months.	GSH levels, CD4 count and body weight.	Increase in GSH levels, no differences in CD4 count or body weight.	Whey protein increases GSH levels.
Sattler 2008 [79]	United states, 59 individuals receiving ART.	Daily whey protein (40 g) for 12 weeks.	Weight, lean body mass	No difference in weight or lean body mass, but significant increase in CD4 count.	Whey protein increases CD4 count.

NAC = N-acetyl cysteine, GSH = gluthathione

## 10. Probiotics

Recent findings suggest that the mucosal immune system is rapidly and preferentially [80] depleted of CD4 cells during initial HIV infection. This is associated with an enhanced intestinal epithelial permeability, as evidenced by increased levels of lipopolysaccharides (LPS) [81]. Plasma LPS levels correlate with systemic inflammation, which may drive HIV replication and consequently, progression of the disease [82]. Also, HIV is associated with increased markers of intestinal inflammation [83] and has been shown to elicit a Th-2 skewed immune response, which may inhibit an effective antiviral response [84,85]. The gut microbiota of people living with HIV is dramatically different than that of healthy populations. The former group have a notable reduction of both lactobacilli and bifidobacteria, while pathogenic species, such as *Candida albicans* and *Pseudomonas aeruginosa*, are more prominent [83,86]. 

Probiotics are defined as “live micro-organisms, which, when administered in adequate amounts, confer a health benefit on the host” [87]. Studies indicate that specific probiotic strains may reduce epithelial permeability [88,89,90,91], down-regulate systemic and mucosal inflammation [92,93,94], and regulate “allergy-like” Th-2 responses [95]. Therefore, specific probiotic strains may potentially ameliorate HIV-induced changes to the mucosal and systemic immune systems. 

To test this hypothesis, a small RCT (n = 77, of which 42 were receiving ART) was conducted in Brazil amongst children living with HIV. After daily supplementation of candidate probiotic containing strains of *Bifidobacterium bifidum* and *Streptococcus thermophilus* (2.5 × 10^10^ colony forming units) for two months, the CD4 count among the treatment group increased *+*118 cells/μL *versus* a decrease of −42 cells cells/μL among the placebo group (p = 0.05). However, the viral load was not measured, and no reduction in diarrhea incidence was shown [96]. Encouraging results were also reported in a small RCT (n = 24) among HIV infected women in Nigeria (table 7). After four weeks of *Lactobacillus rhamnosus* GR-1 and *Lactobacillus reuteri* RC-14 supplementation, the probiotic group experienced an increase of *+*6.7 CD4 cells/μL, compared to a decrease of −2.2 CD4 cells/μL among the placebo group (p = 0.05) [97]. Although preliminary results are encouraging for probiotic supplementation for people living with HIV, the trials conducted to date have not had sufficiently large sample sizes or length of follow-up to substantiate the evidence for increases in CD4 count or clinical parameters of HIV progression. 

## 11. Prebiotics

A prebiotic is defined as “a selectively fermented ingredient that allows specific changes, both in the composition or activity, in the gastrointestinal microflora that confers benefits upon host well-being and health” [98]. Specific prebiotic formulations have been shown to reduce intestinal permeability [99], down-regulate mucosal inflammation [100], and enhance a systemic Th1 immune profile [101,102]. A recently conducted RCT (n = 57) was the first to show the potential of prebiotic supplementation amongst a population living with HIV. Participants were randomized to receive a placebo, a single dose (15g), or a double dose (30g), of a specific mixture of galacto-oligosacharides (GOS), which are long-chain fructans and pectin-derived oligosaccharides. The prebiotic intervention resulted in increased bifidobacterial levels and reduced numbers of the pathogenic *Clostridium histolyticum* cluster [103]. Furthermore, the prebiotic intervention was associated with reduced CD4*+*/CD25*+* cell activation and an improved Natural Killer (NK) cell activity. Effects on CD4 count or viral load were not reported [104]. Prebiotics can be supplemented with probiotics (once combined, they are referred to as synbiotics). A large RCT in Malawi (n = 795) testing the effect of SynbioticForte 2000 (Table 5) on malnutrition also included a proportion of HIV infected children (n = 361). Although there was no improvement in nutritional cure, there was an overall reduction in outpatient mortality, including a trend towards a reduced mortality among the subgroup of HIV infected children [105]. In conclusion, the application of prebiotics to reverse HIV induced intestinal changes has considerable potential, and further design and testing of products specifically for HIV is warranted.

**Table 7:** Randomized, placebo controlled trials on probiotics and prebiotics and HIV progression.

Reference	Population, in- and exclusion criteria	Intervention and follow-up	Primary outcomes	Major findings	Conclusions
Trois 2007 [96]	Brazil, 77 children (42 receiving ART).	Daily *B. bifidum* and *S. thermophilus* (2.5 x 10^10 ^CFU/ml) for two months.	CD4 count and diarrhea prevalence.	Increase in CD4 count, no reduction in diarrhea prevalence.	The candidate probiotic strains may increase CD4 count among children.
Land 2008 [104]	Italy, 57 ART naïve individuals.	Daily prebiotic 15 g or 30 g for 12 weeks^1^.	NK cell activity and CD4 cell activation.	Increased NK cell activity, reduced CD4 *+*/CD25*+* cell activation and beneficial effect of intestinal flora.	Prebiotics may reduce HIV associated hyper-immune activation and enhance anti-viral response.
Anukam 2008 [97]	Nigeria, 24 ART naïve women	Daily *L. rhamnosus* GR-1 and *L. reuteri* RC-14 (>1.0 x 10^7^ CFU/mL) for 4 weeks.	CD4 count and resolution of diarrhea	Increase in CD4 count and faster resolution of diarrhea.	The probiotics may increase the CD4 count and reduce the length of diarrheal episodes.
Kerac 2009 [105]	Malawi, 361 ART naïve children with malnutrition.	Daily Synbiotic Forte 2000^1^ for 9 months.	Nutritional cure, mortality.	No difference in nutritional cure but trend towards reduced mortality.	Synbiotic may reduce mortality.
Lange 2009 [106]	Various countries, 340 ART naïve individuals.	Daily prebiotics, NAC, bovine colostrum, omega-3 PUFA’s and micronutrients for one year^2^.	CD4 count and viral load.	Increase in CD4 count, no decrease in viral load.	Intervention increases CD4 count and may delay the progression of HIV.

CFU = colony forming unit, NAC = N-acetyl cysteine, NK = natural killer

1. Synbiotic included, *Pediococcus pentosaceus*, *Leuconostoc mesenteroides*, *Lactobacillus paracasei ssp paracasei*, and *Lactobacillus plantarum* (10¹¹ colony-forming units of bacteria total) and four prebiotic fermentable bioactive fibers (2.5 g of each per 10¹¹ bacteria) (oat bran [rich in β-glucans], inulin, pectin, and resistant starch).

2. Formula included short-chain Galactooligosaccharides (scGOS), long-chain Fructooligosaccharides (lcFOS) and Acidic Oligosaccharides from pectin hydrolysate (AOS) (ratio 9,1,10), NAC, bovine colostrum, omega-3 PUFA’s and micronutrients.

## 12. Conclusions

The beneficial effects of supplementation with vitamins B, C, E, and folic acid to HIV patients have been firmly established, and enough evidence exists to recommend the Tanzanian supplement to pregnant women with HIV in developing countries. The potential beneficial effects of this multivitamin supplement among HIV patients who are being treated with ARTs and among male HIV patients remain to be determined. 

Among children living with HIV, evidence indicates that vitamin A is beneficial in reducing morbidity and mortality in developing countries. Further large RCTs with HIV progression and mortality as primary outcomes are warranted to substantiate these findings. Among pregnant women with HIV, there is little evidence to recommend vitamin A supplementation. The potential increase in MTCT found in one study due to vitamin A supplementation warrants caution for its use among this population. 

Only one RCT has been conducted to date to assess the effect of vitamin D on HIV progression. The vitamin did not show apparent benefits. This might have been due to an insufficient dose or a relatively small sample size of the sub-population co-infected with HIV. Therefore, a large RCT with HIV progression as the primary outcome and a potentially different dose would be of great value.

A small RCT on zinc has provided some evidence of beneficial effects among patients treated with ART. However, a study among ART-naïve pregnant women did not show similar results. In contrast, supplementation of zinc was associated with an increased risk of wasting and anemia. Until these concerns are addressed by further research, caution is warranted for the use of zinc among people living with HIV. 

No apparent adverse effects on HIV progression have been detected with low-dose iron supplementation. However, the studies conducted do not exclude more subtle adverse effects of iron supplementation on HIV progression. Therefore only an RCT with a sufficient sample size to detect more subtle changes would be able to rule out potential adverse effects of iron.

Small studies have indicated beneficial effects of selenium, NAC, or whey protein on HIV progression. However, these findings need to be confirmed by larger, well designed RCTs. Furthermore, probiotics and prebiotics have limited preliminary data for their use among people living with HIV and further research is warranted.

An integrated approach that combines micronutrients with the various functional food components may have the greatest potential to delay HIV progression. A large multicenter trial, recently conducted in Europe, provides a prime example for this approach. The RCT among ART naïve patients (n = 370) showed that a novel formulation (not fully disclosed, but including prebiotic oligosaccharides with NAC, bovine colostrum, omega-3 poly-unsaturated fatty acids and a combination of vitamins and minerals) slowed down the decline of the CD4 count with 28 cells/μL in the intervention group, compared to −68 cells/μL among the placebo group after one year of supplementation (p < 0.05) [106]. This is very encouraging, although the formulation would be expensive and perhaps not so readily available to people living in developing countries. 

It should be emphasized that an adequate micronutrient intake among people living with HIV is best achieved through an adequate diet [7]. On the other hand, various interventions have been shown to have beneficial effects in dosages not feasible to be obtained from a diet or not naturally present in high concentrations in food. Therefore, in addition to a diet-based approach, the development of functional food interventions specifically for people living with HIV are much needed.
